# Introduction of a method to calculate cumulative age- and gender-specific lifetime attributable risk (LAR) of cancer in populations after a large-scale nuclear power plant accident

**DOI:** 10.1371/journal.pone.0228549

**Published:** 2020-02-05

**Authors:** Christopher Rääf, Nikola Markovic, Martin Tondel, Robert Wålinder, Mats Isaksson

**Affiliations:** 1 Medical Radiation Physics, Department of Translational Medicine, Lund University, Malmö, Sweden; 2 Department of Radiation physics, Institute of Clinical Sciences, Sahlgrenska Academy University of Gothenburg, Gothenburg, Sweden; 3 Occupational and Environmental Medicine, Department of Medical Sciences, Uppsala University, Uppsala, Sweden; 4 Occupational and Environmental Medicine, Uppsala University Hospital, Uppsala, Sweden; University of Colorado Denver Skaggs School of Pharmacy and Pharmaceutical Sciences, UNITED STATES

## Abstract

The effect of age and gender in risk estimates related to long-term residence in areas contaminated by nuclear power plant fallout was evaluated by applying the lifetime attributable risk (LAR) concept to an existing exposure model that was previously used for cumulative effective dose estimates. In this study, we investigated the influence of age distribution on the number of cancer cases by applying five different age distributions from nuclear power–producing countries (India, Japan, South Korea, and the United States), and Egypt because of intentions to develop nuclear power. The model was also used to estimate the effective dose and gender-specific LAR as a function of time after fallout for the offspring of the population living in ^137^Cs fallout areas. The principal findings of this study are that the LAR of cancer incidence (excluding non-fatal skin cancers) over 70 y is about 4.5 times higher for newborn females (5.4% per MBq m^-2^ of initial ^137^Cs ground deposition) than the corresponding values for 30 y old women (1.2% per MBq m^-2 137^Cs deposition). The cumulative LAR for newborn males is more than 3 times higher (3.2% versus 1.0% per MBq m^-2 137^Cs deposition). The model predicts a generally higher LAR for women until 50 y of age, after which the gender difference converges. Furthermore, the detriment for newborns in the fallout areas initially decreases rapidly (about threefold during the first decade) and then decreases gradually with an approximate half-time of 10–12 y after the first decade. The age distribution of the exposed cohort has a decisive impact on the average risk estimates, and in our model, these are up to about 65% higher in countries with high birth rates compared to low birth rates. This trend implies larger average lifetime attributable risks in countries with a highly proportional younger population. In conclusion, the large dispersion (up to a factor of 4 between newborns and 30 y olds) in the lifetime detriment per unit ground deposition of ^137^Cs over gender and age in connection with accidental nuclear releases justifies the effort in developing risk models that account for the higher radiation sensitivity in younger populations.

## Introduction

Conventional radiological risk assessments for stochastic radiation effects (predominantly radiation-induced cancer) resulting from nuclear reactor accidents or other accidental releases of radionuclides entail the use of the effective dose from various exposure pathways (e.g., [1–4]). The concept of effective dose was introduced in 1991 by the International Commission on Radiological Protection [5] and was further developed in 2007 [6]. The effective dose is suitable for comparing the risk that one or more representative fictive individuals develop cancer in various scenarios. It can be considered for use in an optimization tool in planned exposure situations as well as an input for managing mitigation measures in existing exposure situations and in radiological and nuclear emergencies. The effective dose to a representative individual can be translated into a measure of detriment, indicating the absolute risk of attaining a radiation-induced cancer, by multiplying the effective dose with a detriment-adjusted nominal risk coefficient. According to the ICRP [6], a coefficient of about 0.05 per Sv (representing roughly a 5% absolute risk of attaining a radiation-induced cancer per unit effective dose in the case of uniform whole-body irradiation) is used as an average for exposed members of the public, regardless of sex distribution.

Lifetime attributable risk (LAR) coefficients, specified according to radiation-exposed organ, gender, and age at exposure, were presented by the United States National Research Council’s Committee to Assess Health Risks from Exposure to Low Levels of Ionizing Radiation (BEIR) in report VII [7] and further developed by the United States Environmental Protection Agency (EPA) in 2011 [8]. The LAR coefficients for the different organs were presented in terms of excess number of radiation-induced cancer cases and cancer fatalities per 10,000 individuals and unit organ-absorbed dose. The EPA risk coefficients are based on epidemiological findings from, e.g., Japanese A-bomb survivors, combined with baseline cancer-incidence rates in the North American population. The LAR concept is primarily intended for external exposures but has also been applied for internal exposures (e.g., [9–11]). A WHO report [9] used exposure data to estimate the lifetime organ doses to the general population, summed over both internal and external exposure, after the Fukushima Dai-ichi nuclear power plant (FDNPP) accident in Japan in 2011. In turn, this was used to find the lifetime attributable risk for various age cohorts. Yasuda (2018) [12] used LAR instead of the average effective dose to estimate the long-term detriment resulting from the ground deposition of ^134^Cs and ^137^Cs after the FDNPP accident. The approach provided some age- and gender-specific features of the absorbed dose per unit ground deposition and (apparently) assumed a homogeneous dose distribution in all risk organs from the external exposure of ^134^Cs and ^137^Cs.

Isaksson et al. (2019) [13] proposed a model that sums up the contributions from external and internal exposures that are likely under different scenarios related to nuclear power plant (NPP) releases. In these scenarios, large regions are contaminated by the ground deposition of long-lived fission products such as ^137^Cs. This model can also be extended to calculate absorbed doses to specific organs, since the distribution of the internal contamination of fission products can be modelled. For the NPP releases, both the lifetime internal and external absorbed dose are predominantly governed by ^134^Cs and ^137^Cs. It is generally assumed that radiocaesium is distributed uniformly in the body when transferred through the food chain to humans, and it mainly accumulates in cell-rich tissues such as muscle (e.g., ICRP, [14]). This implies that long-term internal exposure will result in relatively uniform organ exposures, except skin and other tissues positioned close to the body surface that will exhibit lower absorbed doses than internal organs (Snyder et al., 1975[15]). Leggett et al. (1984) [16] translated the uniform distribution of radiocaesium in humans into a model of the average whole-body dose and corresponding effective dose. A simplified expression, later derived from Legget’s model by Falk et al. (1991) [17], expresses the effective dose as a function of body weight and thus also of age. Albeit that this age and body size application of effective dose is obsolete today according to the recommendations by ICRP 103 [7], the expressions by [17] can still be used for determining the average absorbed dose to organs if the radiocaesium is distributed homogenously in the body. For body weights of 70 kg the formula by [17] agree numerically with the corresponding internal absorbed whole body dose computed by [15] for a homogenous distribution in the body. There are also age-specific differences in organ absorbed doses from external exposures, mainly due to the variation in body height with age that increases the distance between the ground deposition and the risk organs (an age-dependent factor for external thyroid doses is given e.g., [18]). Taking these features into account in the model enables a more extensive illustration of how the absorbed dose, vary with age and gender. After age- and gender-specific organ doses from internal and external exposures have been computed, it is also possible to apply age- and gender-dependent risk estimates in terms of LAR, taken from, e.g., the EPA report [8].

The aim of this study is to assess age- and gender-specific aspects of radiological consequences, in terms of cancer incidence in populations with different age distributions, from different scenarios related to a major NPP release. The study compares the anticipated detriment due to the cumulated effective dose over 70 y to a reference person (and the associated detriment-adjusted nominal risk coefficient of 0.05 Sv^-1^) and the lifetime attributable risk (average individual probability of radiation-induced cancer incidence) over the same time period. Moreover, the gender- and age-dependency in the estimated risk of total cancer incidence is discussed at the population level with a 70 y perspective. The aim is also to use the model to estimate risk in future offspring of inhabitants living in non-mitigated areas contaminated by accidental NPP releases. Finally, the impact of different age distributions on the detriment estimates, in terms of average committed effective dose over 70 y and the corresponding LAR value for total cancer incidence, is investigated by applying typical examples of age distributions encountered in nuclear energy–producing countries (India, Japan, South Korea, and the United States of America) and a country (Egypt) that intends to build nuclear power for domestic electricity production.

## Materials and methods

### Description of an exposure model for residents in a contaminated area: Cumulative effective dose to reference person

Our previous study presented a model of the long-term external and internal radiation doses to inhabitants living in an area affected by nuclear fallout [13]. The model assumes that the contributions from both external and internal exposure to short-lived fission products and the neutron activation product, ^134^Cs, can be related to the initial fallout of the long-lived fission product, ^137^Cs, in terms of the equivalent plane source deposition of ^137^Cs, *A*_*esd*_ (Bq m^-2^). The advantage of relating all these effective dose contributions to *A*_*esd*_ is that this quantity is relatively easy to determine shortly after the accident using mobile gamma spectrometry (airborne or car-borne systems). In turn, this enables extensive geographical mapping of the fallout and subsequent predictions of the long-term averted radiation dose, depending on the geographical location and mitigating actions. Alternatively, the total ground deposition density, *A*_*tot*_ (Bq m^-2^), of ^137^Cs can be determined from extensive soil sampling, which was the case for, e.g., the Chernobyl fallout in Central Europe and in the Fukushima prefecture after the NPP accident in 2011 (e.g., [19–20]). Since the latter method appears to be more globally prevailing, the models presented here are thus expressed in terms of the initial total ground deposition density, *A*_*tot*_. Furthermore, it was assumed that the incorporated radiocaesium will behave as the alkali metal potassium, which is homogeneously distributed in the body, and hence results in a uniform activity concentration of ^134^Cs and ^137^Cs (Bq kg^-1^) in all organs and cellular tissues in exposed individuals [14].

From the studies described in [13] and [21], a condensed expression of the cumulated effective dose, *CED*(*t*_*acc*_, *A*_*tot*,*loc*_, *A*_*tot*,*reg*_), incurred by the population residing at a location with a local ^137^Cs deposition of *A*_*tot*,loc_ (Bq m^-2^), situated in a region (>1000 km^2^) with an average regional ^137^Cs deposition of *A*_*tot*,reg_, can be obtained in mSv, as in Eq (1).

$$\begin{array}{l} CED\left(t_{acc},A_{tot,loc},A_{tot,reg}\right)=A_{tot,loc}\cdot S_{decont}\cdot d_{Cs}\cdot \emptyset_{K/H}(600\mathrm{keV})\cdot C_{E/K}\cdot \int_{t_{0}}^{t_{acc}}r(t)\cdot f_{snow}\cdot \\ \left(f_{out}+\left(1-f_{\mathrm{out}}\right)\cdot f_{\mathrm{shield}}\right)\cdot dt+A_{\mathrm{tot},\mathrm{reg}}\cdot T_{\mathrm{ag},\max}\cdot S_{\mathrm{aliment}}\int_{t0}^{t_{\mathrm{acc}}}(\left(1-e^{\frac{-ln(2)}{t_{1}}\cdot t}\right)\cdot (c_{1}\cdot e^{\frac{-ln(2)}{t_{2}}\cdot t}+c_{2}\cdot \\ e^{\frac{-\ln(2)}{t_{3}}\cdot t}))\cdot f_{\mathrm{sex}}\cdot \left(e_{Cs-137}(w)+FR\cdot e^{\left(\frac{\ln2}{T_{½,Cs-137}}-\frac{\ln2}{T_{½,Cs-134}}\right)\cdot t}\cdot e_{Cs-134}(w)\right)\cdot dt \end{array}$$ (Eq 1)

The remaining variables in this expression are briefly described in Table 1. To attain values that are representative for both men and women body weights of w = 70 kg were used for the internal dose contributions from radiocaesium. The model does not account for the corresponding effective dose from the internal exposure of short-lived radionuclides, such as ^131^I, ^132^I, and ^132^Te, through inhalation and contaminated foodstuffs. These make an important contribution to the first-year effective dose but only a small contribution to the *CED*(70y). If major remedial actions are undertaken, such as stabling of dairy cattle and the distribution of stable iodine, this dose contribution is negligible in terms of lifetime effective dose. The estimated contribution to the total committed effective dose from consuming ^131^I in milk is about 10 mSv at a ground deposition of 1 MBq m^-2 137^Cs [13], compared to about 140–1490 mSv for the 50 y committed effective dose from internal exposure of ^134^Cs and ^137^Cs and external exposure during the same period of time. The influence from iodine inhalation and cloud shine will also be modest in comparison with the 50 y committed dose, as long as proper sheltering is done as demonstrated by the thyroid dose modelling from the Chernobyl fallout for residents in Sweden [18].

**Table 1:** Parameter values used for calculation of effective dose from a nuclear power plant fallout (Eq (1)).

Parameter	Description (unit)		
*A*_*tot*,*loc*_(*x*,*y*)	Average local deposition at the dwelling coordinate (symbolized with x and y) of ^137^Cs (kBq m^-2^), decay corrected to the time of the fallout event. This quantity is often obtained through airborne gamma spectrometry mapping used in, e.g., geological surveys. Maps of fallout can then be made with relatively high spatial resolution (e.g., 200 by 200 m^2^), as has been done by, e.g., SGU in Sweden [22].		
*A*_*tot*,*reg*_	Regional average of *A*_*tot*,*loc*_ (kBq m^-2^). An average over an area representing a region defined in terms of administrative or economical relevance for the local population, and from which the main part of the ingested local food by the residents in the area originates. In Sweden, such regions are defined over areas that range between 3,000 to 100,000 km^2^ in size. These regions may substantially differ between countries and may be smaller in countries with a high degree of local food production.		
*d*_*Cs*_	Empirical correlation factor (= 1.02 mSv y^-1^/kBq m^-2^) between the so-called surface equivalent deposition, *A*_*esd*_, of fresh fallout from the Chernobyl accident and the ambient dose rate 1 m above ground, taken from [21]. The expression in Eq (1) was originally derived for surface equivalent deposition, *A*_*esd*_, which is defined as the areal activity concentration of a plane source that will cause the same dose rate 1 m above the surface as the actual depth-distributed areal activity concentration. Deposition maps of *A*_*esd*_ after the Chernobyl fallout were widely used in Sweden due to the straightforwardness of airborne measurements instead of performing laborious soil samplings for the assessment of total activity deposition, *A*_*tot*_. Empirically, it was found that the ratio between the total Chernobyl ^137^Cs deposition density in Sweden and *A*_*esd*_ was 1.6, based on a measurement survey conducted by [23], and it can be assumed that this value is relatively representative for wet deposited fresh fallout of radiocaesium, where precipitation has transported the radiocaesium at least a few centimetres into the soil. In this study, however, the factor 1.6 is now incorporated into *d*_*Cs*_, so that the parameter relates to the ambient dose rate per unit total activity deposition, and thus assumes the value 1.02·(1/1.6) = 0.636 mSv y^-1^/kBq m^-2^.		
∅_*K*/*H*_(600*keV*)	Ratio between air kerma rate and ambient dose equivalent rate 1 m above ground for an infinite uniform surface deposition of gamma emitters with photon energy 600 keV (mGy mSv^-1^). A value of 0.83 has been used, taken from [24].		
*C*_*E*/*K*_	Ratio between effective dose rate and air kerma rate [25], given in mSv mGy^-1^. A value of 0.82 was used for rotational geometry by [21] to represent a deposition of gamma emitters with a mean primary energy of 600 keV for a rotational symmetric irradiation geometry. However, in this work, this number is replaced with a value of 0.73 taken from [25], which better represents the conversion between effective dose and air kerma rate for an irradiation geometry of a plane-surface deposition. This value has been somewhat adjusted for the slight difference in the ratio of effective dose rate and air kerma rate values between the old reference from 1997[26] and the newer one from 2010[25].		
*f*_*snow*_	Snow cover shielding factor (unity) averaged over the whole year for ambient dose rate 1 m above ground. In our study, no snow cover was considered, and *f*_*snow*_ was thus set to unity.		
*r*(*t*)	Time-dependent function describing the decrease in external ambient dose rate 1 m above ground, normalized to the maximum initial dose rate following a nuclear power plant fallout corresponding to a Chernobyl-like wet deposition at remote locations from the release point. Apart from external gamma contribution from ^134^Cs and ^137^Cs, corresponding contributions from gamma emitters, such as ^131^I, ^132^I, ^132^Te, and ^140^Ba, are included [21]. A time-dependent function composed of four components was taken from [21], with time constants expressed in terms of y^-1^. *r*(*t*) = 0.96·e^-36.9·t^+0.10823·e^-2.45·t^+0.0796·e^-0.668·t^+0.0314·e^-0.126·t^.		
*f*_*out*_	Time fraction spent outdoors for an individual residing in a temperate climate zone. Typical values range between 0.1 and 0.2 for Northern European populations [27–31]. A value of *f*_*out*_ = 0.2 was used in this work.		
*f*_*shield*_	Shielding factor for indoor stay, ranging between 0.10 and 0.4 for Northern European houses [32]. A value of *f*_*shield*_ = 0.4 was used in this work.		
*t*_*acc*_	Time over which the radiation exposure is integrated (y).		
*T*_*ag*,*max*_	Maximum transfer factor aggregated over all radioecological transfer pathways. This parameter determines the magnitude of the time-dependent transfer, *T*_*ag*_(*t*) (Bq kg^-1^)/(kBq m^-2^), from regional-average ground deposition to whole-body concentration of ^134,137^Cs in residents. Analogous with the factor *d*_*Cs*_, this factor was calculated with respect to *A*_*esd*_ and is here adjusted downwards a factor 1.6 from the values given in [13]; thus, it is expressed with respect to the total ^137^Cs deposition, *A*_*tot*_. The value of *T*_*ag*,*max*_ thus varies from 6.7 in the general population to ca. 20 Bq kg^-1^/(kBq m^-2^) for hunters and more than 115 Bq kg^-1^/ (kBq m^-2^) for reindeer herders in Sweden. The value for the general population of 6.7 Bq kg^-1^/(kBq m^-2^) was used in this work.		
*w*(*age(t*_*0*_*+t)*)	Body mass (kg) as a function of age. A curve fit of data taken from [33] has been done to yield the following expressions:		
	Age	Weight (Females) (kg)	Weight (Males) (kg)
	age<20 y	-0.0000057·age^6^+0.000552·age^5^- 0.0199·age^4^+0,3191·age^3^-2.1579·age^2^+7.4423·age+3.9529	-0.0000021·age^6^+0.0002623·age^5^-0.011799·age^4^+0.2305·age^3^-1.8759·age^2^+8.0766·age+3.8872
	Age>20 y	63	78
*t*_*1*_, *t*_*2*_ and *t*_*3*_	Time constants of radioecological transfer depending on type of population. Values used here are *t*_*1*_ = 1.0 y, *t*_*2*_ = 0.75 y, and *t*_*3*_ = 15 y. Values for other types of populations can be found in [13].		
*c*_*1*_ and *c*_*2*_	Coefficients of amplitude of radioecological transfer depending on type of population. Values used here refer to urban populations in Scandinavia and are *c*_*1*_ = 1.0 and *c*_*2*_ = 0.10. Values for other types of populations can be found in [13].		
*T*	Time in y.		
*T*_*1/2*,*Cs-137*_	Physical half-life of ^137^Cs: 30.2 y.		
*T*_*1/2*,*Cs-134*_	Physical half-life of ^134^Cs: 2.06 y		
*FR*	Isotopic ratio ^134^Cs/^137^Cs at the time of initial fallout. A value of 0.56 was reported by [34] for the Chernobyl NPP fallout, and 1.1 for the Fukushima Dai-ichi NPP release [35]. The value for *FR* used here = 0.56.		
*f*_*sex*_	Empirical factor accounting for the lower observed radiocaesium concentration per unit body mass in women compared with adult males [36]. *f*_*sex*_ = 0.61 for females aged >20 y; *f*_*sex*_ = 1 for males at all ages and females <20 y. For cumulative effective dose computation the mean of adult men and women was used in Eq 1 with a corresponding average of *f*_*sex*_ = 0.81.		
*e*_*Cs*−137_	The effective dose rate conversion factor (mSv y^-1^/(Bq kg^-1^)) taken from [17], based on the biokinetic models by [16]. This is expressed as *e*_*Cs-137*_(w) = 0.0014∙*w*(*age*(*t*))^0.111^, where the factor 0.0014 is a curve fit constant and *w*(*age*) (kg) is the mean body weight of an individual at a certain age. It is assumed that this quantity is numerically equal to the absorbed dose rate per unit activity concentration in the body (see also Rääf et al., 2019) [22].		
*e*_*Cs*−134_	The effective dose rate conversion factor (mSv y^-1^/(Bq kg^-1^)) taken from [17], based on the biokinetic models by [16].This is expressed as *e*_*Cs-134*_(w) = 0.00164∙*w*(*age*(*t*))^0.188^, where the factor 0.00164 is a curve fit constant and *w*(*age*) (kg) is the mean body weight of an individual at a certain age. It is assumed that this quantity is numerically equal to the absorbed dose rate per unit activity concentration in the body (see also [18]).		
*S*_*decont*_	Factor representing the ratio between the ambient dose rate in the area after and before a decontamination procedure. Since the calculations in this study refer to unmitigated conditions with no countermeasures carried out, *S*_*decont*_ is by definition set to unity.		
*S*_*aliment*_	Factor representing the relative decrease in proportion to the standard radioecological transfer factor of foodstuffs brought on by various countermeasures. Since the calculations in this study refer to unmitigated conditions with no countermeasures carried out, *S*_*aliment*_ is by definition set to unity.		

### Description of risk model of fictive residents in a contaminated area: Cumulative absorbed organ and whole-body dose

The organ-absorbed doses (mGy) can be expressed in a modified Eq (1), considering the specific organ dose coefficients. This expression can be simplified if it is assumed that both short-lived gamma emitters as well as ^134^Cs and ^137^Cs, on average, emit photons with energy 600 keV (as argued in [21]). Moreover, the external contribution to the absorbed dose in an organ can be converted from the air kerma rate by inserting an organ-specific coefficient, *k*_*SEQ*,*Organ*,*ext*_ (Gy Gy^-1^), taken from [26]. An age-dependent factor, *k*_*SEQ*,*K*_, accounting for the body stature dependence in *k*_*SEQ*,*Organ*,*ext*_, is introduced. The sum of the external and the internal contributions to a specific organ dose, *D*_*org*,*sex*_ (mGy), will then be (Eq 2):

$$\begin{array}{l} D_{org,sex}\left(t_{acc},age,A_{tot,loc},A_{tot,reg}\right)\\ \;=A_{\mathrm{tot},\mathrm{loc}}\cdot S_{\mathrm{decont}}\cdot d_{Cs}\cdot \emptyset_{K/H}(600\mathrm{keV})\cdot f_{\mathrm{snow}}\cdot \\ \;\cdot k_{SEQ,\mathrm{Organ},\mathrm{ext}}\int_{t0}^{t_{acc}}r(t)\cdot k_{SEQ,K}(age)\cdot \left(f_{out}+\left(1-f_{out}\right)\cdot f_{\mathrm{shield}}\right)dt\\ +A_{\mathrm{tot},\mathrm{reg}}\cdot T_{\mathrm{ag},\max}\cdot S_{\mathrm{aliment}}\int_{t_{0}}^{t_{\mathrm{acc}}}(\left(1-e^{-\frac{-ln(2)}{t1}\cdot t}\right)\cdot \left(c_{1}\cdot e^{\frac{-ln(2)}{t_{2}}\cdot t}+c_{2}\cdot e^{\frac{-ln(2)}{t_{3}}\cdot t})\right)\cdot f_{\mathrm{sex}}\cdot \\ (k_{\mathrm{Organ},\mathrm{int},Cs-137}\cdot e_{Cs-137}(w(age(t))+k_{\mathrm{Organ},\mathrm{int},Cs-134}\cdot FR\cdot e^{(\frac{\ln2}{T_{½},Cs-137}-\frac{\ln2}{T_{½},Cs-134})\cdot t}\cdot \\ e_{CS-134}(w(\mathrm{age}(t)))\cdot dt \end{array}$$ (Eq 2)

where the following parameters, in addition to the ones described in Table 1, are presented in Table 2. The parameters in Tables 1 and 2 are considered in the model for a fictive population living under circumstances mimicking those of a general population in a temperate climate.

**Table 2:** Parameter values used for calculation of organ-absorbed doses (Eq (2)).

Parameter	Description (unit)
*k*_*SEQ*,*Organ*,*ext*,_	Organ-specific absorbed dose rate per unit kerma rate 1 m above ground for an adult of gender female (F) or male (M). Values for the organs related to cancers specified in EPA ([8]) are given as gender-specific *k*_*SEQ*,*Organ*,*ext*_ (Gy Gy^-1^) have been taken from [26]. In our study, the coefficient for colon is used to represent the whole body: 0.686 for males and 0.708 for females, respectively.
*k*_*SEQ*,*K*_(age(*t*))	Age-dependent organ-specific absorbed dose rate per unit kerma rate, normalized against the corresponding value for an adult (female or male, respectively). The age-dependence curve for the thyroid ([18]) is here assumed to be applicable for all organs. *K*_*SEQ*,*K*_(*age*,*t*) = (0.0015·age((*t*))^5^–0.1214·age((*t*))^4^+3.473·age((*t*))^3^–40.28·age((*t*))^2^+136.3·age(*t*)+1233)/1017 for *age*<20 y; and 1 for *age*≥20 y.
*k*_*Organ*,*int*,*Cs-134*_ *k*_*Organ*,*int*,*Cs-137*_	Ratio between organ-absorbed dose and the average whole-body absorbed dose incurred by a uniformly distributed internal contamination of ^134,137^Cs. Ratios for organs specified in EPA [8] are taken from [15]. Here, values for the whole body of 1.0 are used for both caesium isotopes.

In summary, the combined exposure pathways to humans after a radionuclide fallout in a region, excluding initial inhalation, cloud shine, and contributions from internal short-lived nuclides, can in large part be condensed by the expressions of Eqs (1) and (2), where particular organ doses can be deduced from Eq (2). For a discussion of the impact of these factors, refer to [13] and [18].

### Calculation of LAR for total cancer incidence to a fictive person living in a contaminated area

**Fig 1:**
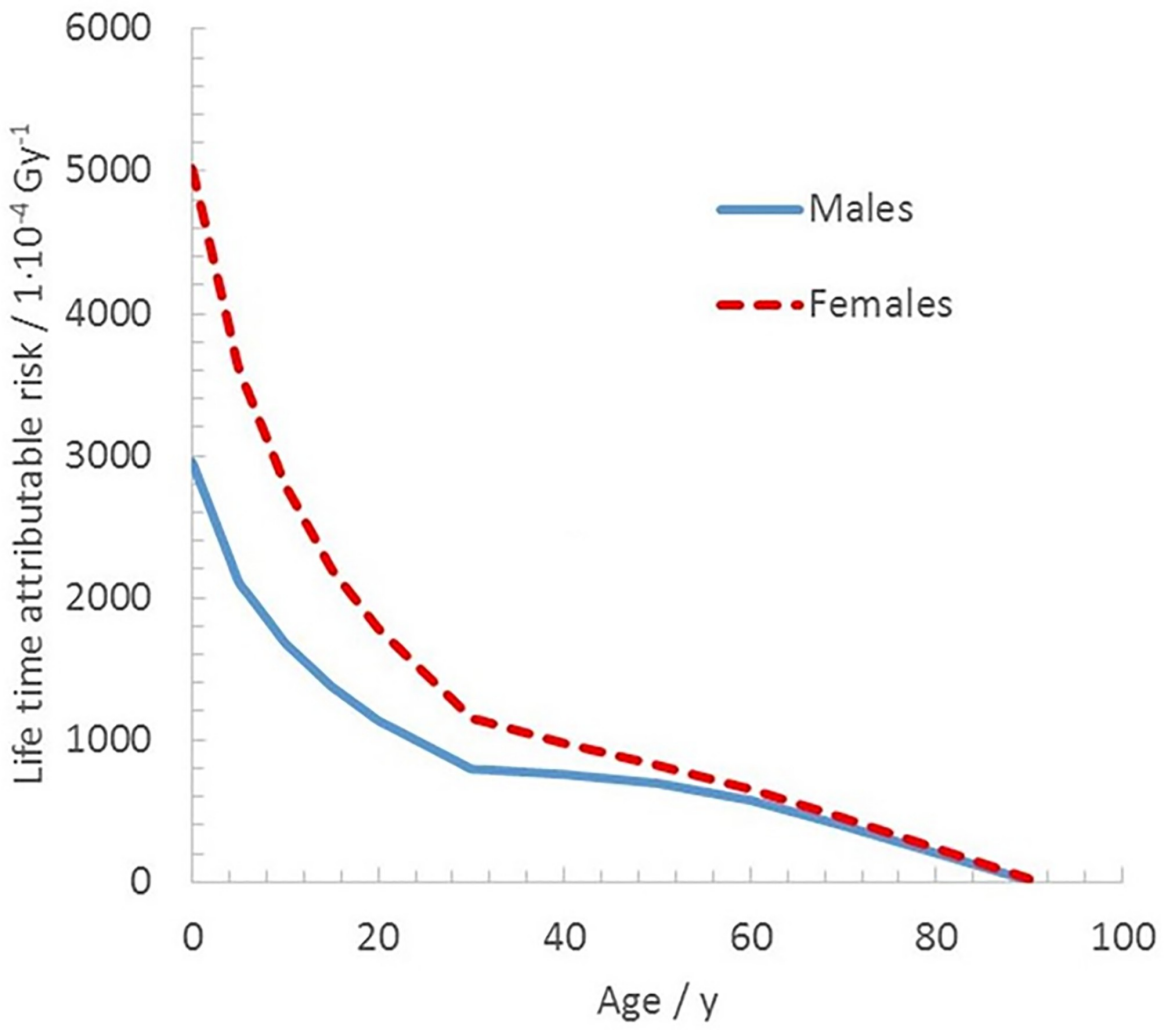
Interpolated continuous age-dependent LAR (10^−4^ Gy^-1^) for total cancers (except non-fatal skin cancers) for protracted radiation exposure as a function of age, taken from EPA (2011).

The absorbed dose rate (mGy y^-1^) for a specific organ, *D*_org_, for a fictive individual of given gender and age at the start of fallout, *t*_*0*_, can be combined with the LAR coefficient (unit: 10,000 cases Gy^-1^) for the organ’s cancer incidence to obtain the corresponding annual rate of LAR. Similarly, the absorbed dose rate averaged over the whole body, *D*_WB_, is used for total cancers (including non-solid neoplasm) in case of whole-body irradiation. EPA (2011) [8] lists LAR coefficients for 13 radiation-induced solid cancer sites, one for the remaining solid cancers, and one for leukaemia. In this study, LAR coefficients for total cancers, taken from Tables 3-12a and 3-12b in the EPA publication [8], are applied for various age and gender categories for five scenarios specified in the next section. The aforementioned LAR values are specifically intended for low-dose or protracted exposures, such as those received by people living in areas affected by radioactive fallout similar to that from the Chernobyl NPP or Fukushima Dai-ichi NPP accidents. Fig 1 plots an interpolation of the resulting LAR coefficients from [8] as a function of age.

The annual effective dose was integrated over *t*_*acc*_ = 70 y, using Eq (1), to obtain the cumulated effective dose, *CED*(70 y). Likewise, annual organ-absorbed doses, *D*_*org*,*sex*_, integrated over the same time period (70 y) using Eq (2) and combined with organ-specific *LAR*_*Org*,*sex*_ coefficients taken from EPA (2011) [8], were calculated. These calculations were performed for fictive residents living in an area with an initial local ground deposition for ^137^Cs of *A*_*tot*,*loc*_ and a regional-average ground deposition of *A*_*tot*,*reg*_, using the expression given in Eq 3.

$$\begin{array}{l} \;CUMLAR_{org,sex}\left(\mathrm{age}\left(t_{0}\right),t_{acc},A_{tot,loc},A_{tot,reg}\right)\\ =\sum_{t0}^{tacc}D_{org,sex}\left(t,age,A_{loc},A_{reg}\right)\cdot LAR_{Org,sex}(age(t)) \end{array}$$ (Eq 3)

where *LAR*_*Org*,*sex*_(age(*t*)) is a monotonically decreasing function of age, interpolated from values tabulated in [8] for 5 y classes. The value of *CUMLAR*_*Org*,*sex*_(age(*t*_*0*_),*t*_*acc*_, *A*_*tot*,*loc*_, *A*_*tot*,*reg*_) thus represents the time-integrated lifetime attributable risk for cancer incidence in a specific organ, attained by the protracted radiation exposure from the onset of fallout at time *t*_*0*_ until time *t*_*0*_*+t*, where *age*(*t*_*0*_) is the age of the person at the time of fallout, *t*_*0*_. The *LAR*_*Org*,*sex*_(*age*(*t*_*0*_*+t*_*x*_+½)) for each year *t*_*x*_ is thus multiplied with the estimated value of the organ-absorbed dose received during that year, *D*_*org*,*sex*_(*t*_*x*_, *age*, *A*_*tot*,*loc*_, *A*_*tot*,*reg*_)), to attain the yearly contribution to *CUMLAR*_*Org*,*sex*_(age(*t*_*0*_),*t*_*acc*_, *A*_*tot*,*loc*_, *A*_*tot*,*reg*_) up to a certain integration period *t*_*acc*_.

This study takes an approach similar to that used by [12], in which only the whole-body dose is considered when calculating the total anticipated detriment in terms of lifetime attributable risk of any cancer over a period up to *t*_*acc*_ = 70 y. The difference compared with [12] is that we consider cancer incidence instead of cancer mortality in order to match the detriment used for the effective dose as defined by ICRP [6]. However, in the ICRP publication, radiation-induced skin cancers have a low weight (tissue weighting factor only 0.01), whereas *LAR*_*skin*,*male*_(age), including non-fatal cancers, accounts for 46% of total LAR for cancer incidence for a newborn boy and is still as high as 6% for a 30 y old male. This makes a comparison between the two concepts a bit inconsistent; therefore, we assessed *CUMLAR* by including total cancer incidence *except non-fatal skin cancers* so that the comparison between *CED* and *CUMLAR* is more consistent.

Furthermore, in epidemiological studies on external exposure to ionizing radiation, the absorbed dose to the colon has often been used to estimate the dose-response relationship with solid cancers. As an example, in a study of INWORKS, the colon dose was used as a proxy to represent the exposure to the rectum, peritoneum, bone/connective tissue, etc. [37]. The colon dose was also used in epidemiological studies on diagnostic X-rays, when organ dose estimates were not available to express excess relative risk for other solid cancers [11]. Thus, instead of using averages of *k*_*SEQ*,*K*_ for the various organs listed in Table 3, the absorbed dose to the colon, *D*_*colon*_, was chosen to better represent the external contribution to the average whole-body dose, *D*_*WB*,*sex*_(age(*t*)). Therefore, in Eq (2), *k*_*SEQ*,*Colon*,*ext*_ was used for the external component, and a value of unity was used for *k*_*Organ*,*int*_ for the internal dose from ^134^Cs and ^137^Cs. This was deduced from the target-source organ relationship given by [15] when estimating the *CUMLAR*(age(*t*_*0*_),*t*_*acc*_, *A*_*tot*,*loc*_, *A*_*tot*,*reg*_) from *t*_*0*_ to *t*_*acc*_ = 70 y. Hence, our *CUMLAR* values refer to calculations where the external contribution to the whole body is calculated based on the absorbed dose to the colon. The *CUMLAR* value for the summed external and internal contributions to all organs in the body will then be denoted as *CUMLAR*_*WB*_(*t*_*acc*_ = 70 y).

**Table 3:** Ratios between organ-specific absorbed dose rate and air kerma rate for a gamma-emitting surface source of 600 keV [26]. Organ-specific ratio between absorbed dose to organ and corresponding whole-body absorbed dose incurred by a uniform distribution of ^134^Cs and ^137^Cs, respectively [15]. N/A = Not available.

Organ specified in Zankl et al. [26]	Associated cancer type (EPA, [8])	*k*_*SEQ*,*Organ*,*ext*_		*k*_*Organ*,*int*,*Cs-137*_	*k*_*Organ*,*int*,*Cs-134*_
		Male	Female	^137^Cs	^134^Cs
Bladder	Bladder	0.696	0.720	1.07	1.24
Skeleton	Bone	0.824	0.804	1	1.05
Stomach	Stomach	0.708	0.731	1	1.14
Colon	Colon	0.686	0.708	1.12	1.27
Liver	Liver	0.711	0.730	1.07	1.19
Lung	Lung	0.762	0.770	1	1.05
Bone marrow	Leukaemia	0.706	0.721	1	1.10
Skin	Skin	0.879	0.883	0.79	0.76
Testes	Residual	0.800	-	1.07	1.19
Thyroid	Thyroid	0.756	0.814	1	1.05
Uterus	Uterus	-	0.665	1.14	1.33
Breast	Breast	-	0.829	N/A	N/A
Ovaries	Ovaries	-	0.706	1	1.05
N/A	Prostate	N/A	N/A	N/A	N/A
Kidney	Kidney	0.723	0.7305	1.07	1.19
Remainder (as defined by ICRP 60 (ICRP, 1991))	Residual	0.716*		1**	1**
Whole body	Total cancers	N/A***	N/A***	1	1

*Refers to gender-averaged values for ten organs: adrenals, brain, upper large intestine (i.e., ascending and transverse colon), small intestine, kidneys, muscle, pancreas, spleen, thymus, and uterus.

**Refers to muscle tissue.

*** The gender-specific values used to represent whole-body exposure for external irradiation will, however, be the values for the colon, in accordance with previous studies related to radiation exposure epidemiology.

When committed dose estimates are performed to represent an individual mean risk among members of the public, an integration time of 50 y is used for adults and an integration up to the age 70 y is used for children [6]. Here, an integration time *t*_*acc*_ = 70 y was employed for fictive individuals for all ages up to 30 y at start of exposure. The cumulated effective dose, *CED* (70 y), given by Eq (1), may then naively be perceived as an individual sample of a population consisting of a never-aging individual of 30 y that lives to the age of 70 y. Therefore, no consideration of life expectancy is needed for the *CED*(70 y) estimates. However, to make a meaningful comparison with detriments calculated from *CUMLAR*(*t*_*acc*_ = 70 y) estimates derived from Eqs (2) and (3), two assumptions regarding survival of the cohort were made. First, as mentioned previously, in the case of protracted and continuous exposure for a fictive individual representing an age and gender cohort, we only considered individuals who have not been diagnosed of, or survived, any cancer induced from radiation exposure during the previous part of the considered time period, up to time *t*_*acc*_. This allows us to compute the maximum radiation risk a person can accumulate up to *t*_*acc*_, given that the person has survived all exposures up to that point.

**Fig 2:**
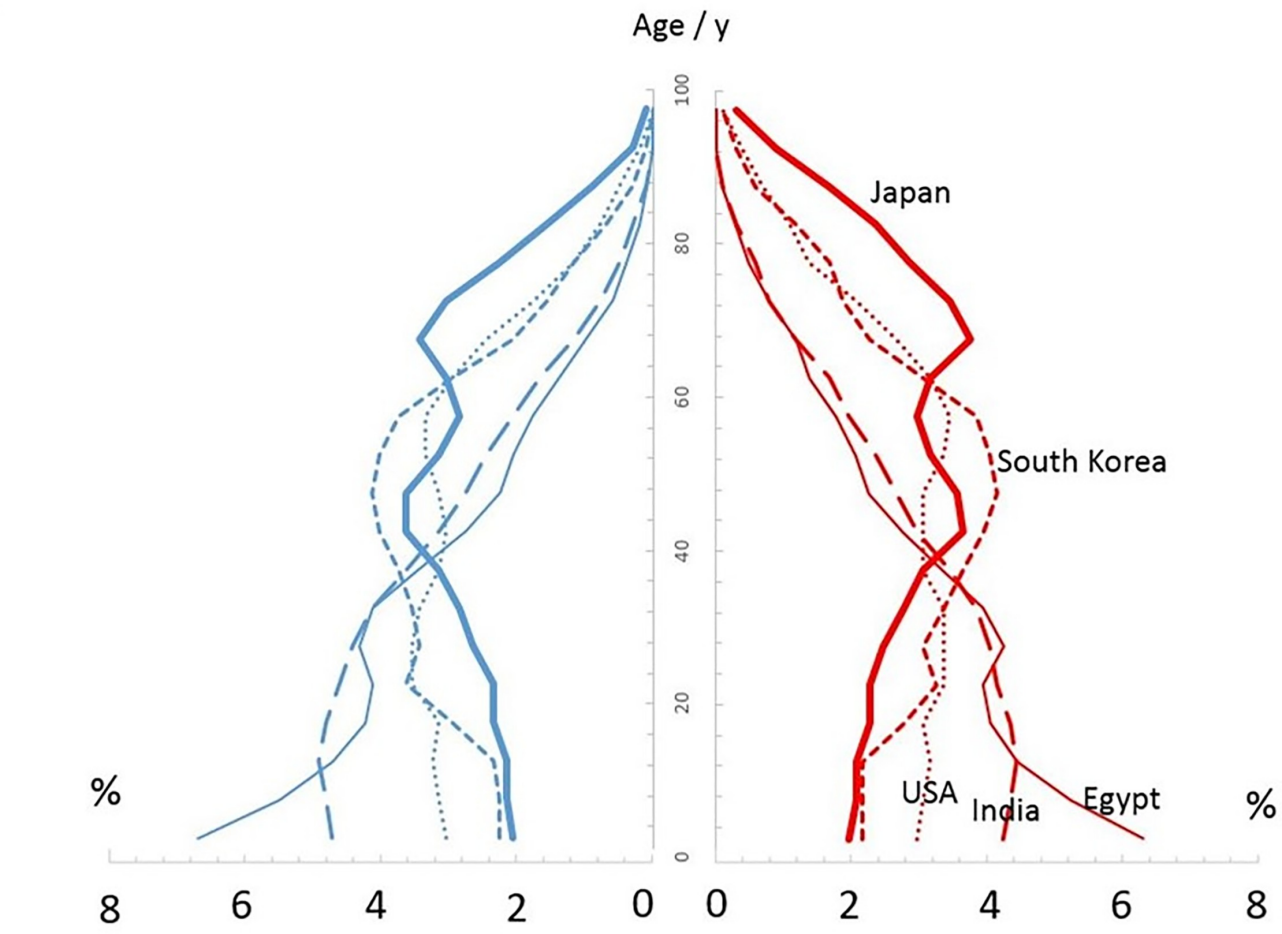
Example of age distributions, *ADW(age)*, taken from UN estimates of Egypt, India, Japan, South Korea, and the United States. Age distribution taken from United Nations (2015).

The significance of the age distribution of an affected population can be illustrated by comparing different characteristic age distributions that are representative of different countries. For a closed cohort without reproduction, a weighted mean can be defined that accounts for the effect of age distribution in the detriment incurred by the radiation dose to a specific organ of the affected population, here denoted as *ADWCUMLAR*_*Org*,*sex*_ in Eq 4. *ADW*_*sex*_(age) is the fraction of a given age cohort for females or males in a population; thus, it is a mathematical representation of the so-called population pyramids (see example in Fig 2). Estimations of such population pyramids (up to age about 100 y old) are available for various countries by several organizations, such as the United Nations [38].

$$\begin{array}{c} ADWCUMLAR_{Org}(age\left(t_{O}\right),A_{tot,loc},A_{tot,reg,,}t_{acc}\\ \begin{array}{l} )=\sum_{age(t0)=0\;y}^{age(t0)=100\;y}ADW(age(t))\cdot \sum_{t0}^{tacc}D_{Org,sex}\left(t,age(t),A_{loc},A_{reg}\right)*LAR_{Org,sex}(\mathrm{age}(t)) \end{array} \end{array}$$ (Eq 4)

In the above expression, the detriment contribution to the population older than 100 y is thus ignored. In summary, Eq 4 describes the age-distribution-weighted average detriment in terms of the time-integrated lifetime attributable risk of radiation-induced cancer incidence in a particular organ. As discussed above, with proper choice of parameters, it may also describe the whole-body exposure and total cancer incidence, *ADWCUMLAR*_WB_(*t*_*acc*_ = 70 y). This quantity can then be compared with the corresponding detriment estimates from the *CED*(70 y) values calculated by integrating over *t*_*acc*_ = 70 y in Eq 1.

### Calculating radiation exposures and detriment for fetuses and offspring of a population living in a contaminated area

The expressions in Eqs (1)–(5) will allow us to make predictions of *CED*(70 y) and *CUMLAR*_*WB*,*sex*_(*t*_*acc*_ = 70 y) for an offspring born at time *t*_*b*_ after the fallout occasion. *CED*(70 y) is calculated by the straightforward time integration of Eq (1), from time *t*_*b*_ to *t*_*acc*_ = *t*_*b*_+70 y. *CUMLAR*_*WB*,*sex*_(70 y) for a newborn individual at time *t*_*b*_ is expressed as Eq 5:

$$\begin{array}{c} {\mathrm{CUMLAR}}_{\mathrm{WB},\mathrm{sex}}\left(\mathrm{age}\left(=0\;\mathrm{at}\;t_{b}\right),A_{\mathrm{tot},\mathrm{loc}},A_{\mathrm{tot},\mathrm{reg}},t_{b}\right)=\sum_{tb}^{tb+70}D_{WB,\mathrm{sex}}(\mathrm{age}(t-\\ t_{b}),A_{loc},A_{reg})*LAR_{WB,Org}\left(\mathrm{age}\left(t_{b}+t_{x}+½\right)\right) \end{array}$$ (Eq 5)

Fetal exposure accrued under gestation can be estimated by assuming that the fetus obtains a dose contribution similar to that of the mother’s uterus, that is, *D*_*Uterus*_(age>20 y) in accordance with Eq (2), integrated over 9 months (= 0.75 y). For illustrative purposes, only the *D*_*Uterus*_(age = 20 y) from the onset of fallout was calculated in order to present the upper limit of radiation dose incurred for a given scenario. The external dose contribution to the fetus is calculated using *k*_*SEQ*,*uterus*,*ext*_ of 0.665. For the internal dose, the corresponding *k*_*Uterus*,*int*,*Cs-134*_ and *k*_*Uterus*,*int*,*Cs-137*_ are set to 1.33 and 1.14, respectively (Table 3). However, the radiation risks associated with exposure to the fetus are not only related to stochastic effects such as cancer, but here, we restrict our risk assessment to cancer. Furthermore, the choice of becoming pregnant while living in a contaminated area has an ethical aspect that is outside the scope of this work.

For illustrative purposes, an overview of the quantities introduced in Eqs (3)–(5) is given in Table 4.

**Table 4:** Quantities defined in Eqs (3)–(5) used for the evaluation of time-integrated (cumulative) lifetime attributable risk estimates.

Quantity	Description (unit)
*LAR*_*Org*,*sex*_ (age(t))	Lifetime attributable risk contribution per unit absorbed organ dose for an individual of age(*t*) at time *t* (probability Gy^-1^). Values of LAR for cancer incidence taken from Tables 3-12a and 3-12b in EPA [8] are used for the protracted exposures in the studied scenarios.
*CUMLAR*_*Org*,*sex*_ (age(*t*_*0*_), *A*_*tot*,*loc*_, *A*_*tot*,*reg*_)	Time-integrated lifetime attributable risk accumulated over time *t*_*0*_ to *t*_*acc*_ for an individual of age(*t*_*0*_) at the onset of exposure (probability).
*ADW*_*sex*_*(age)*	Fraction of an annual age cohort of a specified gender with respect to the whole population (dimensionless). Population pyramids from a number of countries used as case studies are shown in Fig 2, and data for Eqs (5)–(6) retrieved from the United Nations [38].
*ADWCUMLAR*_*Org*,*sex*_(*age*(*t*_*0*_), *A*_*tot*,*loc*_, *A*_*tot*,*reg*_, *t*_*acc*_) (probability)	Age-distribution-weighted time-integrated lifetime attributable risk accumulated over time *t*_*0*_ to *t*_*acc*_ for a given age distribution *ADW*_*sex*_(*age*) (probability).

### Description of the scenarios

The cumulated effective dose, *CED*(*t*_acc_ = 70 y) and *CUMLAR*_*WB*_(*t*_acc_ = 70 y), were calculated for some scenarios of NPP releases described in Table 5, where the basic scenario is a ground ^137^Cs deposition of *A*_*tot*,*loc*_ = *A*_*tot*,*reg*_ = 1 MBq m^-2^ (Scenario A). Study cases in which the recommended reference levels of the annual effective dose are not exceeded were also selected, such as 20 mSv y^-1^ (Scenario B) and 1 mSv y^-1^ (Scenario C); the latter represented a situation where the exposure did not exceed 1 mSv y^-1^ for members of the public, as recommended by the ICRP [6]. Scenario B thus represents a situation that may be the upper limit for considering costly measures, such as extensive decontamination. Due to the decision-making process in an NPP fallout event, Scenario C (*E*_*max*_ = 1 mSv y^-1^) may also be a situation in which the aforementioned countermeasures will still be considered.

**Table 5:** Overview of fallout scenarios, calculated detriment estimates and time frames considered in this model assessment.

Scenario	Categories	Detriment indicator	Time period concerned
A	Initial fallout with *A*_*tot*,*loc*_ = *A*_*tot*,*reg*_ = 1 MBq m^-2^.		
	Newborn male at t = 0	*CED*(70 y)* and *CUMLAR*_*WB*_(70 y)	*t*_*0*_ = 0 to *t*_*acc*_ = 70 y
	Newborn female at *t* = 0		
	30 y old male at *t* = 0		
	30 y old female at t = 0		
	*ADW*_*sex*_(age) as of Egypt, India, Japan, South Korea, and United States of America in 2015, respectively.		
	Newborn females, males and average over both female and males as a function of t after fallout (calculated up t*o t*_*b*_ *=* 60 y)		Cumulating interval in Eq (5) starting from *t*_*b*_ = 0 to *t*_*acc*_ = 70 y; up to *t*_*b*_ = 60 to *t*_*acc*_ = 130 y
	Fetus	*D*_*Uteru*s_(age(>20 y))	*t* = 0 to *t* = 0.75 y (Conception at *t*_*0*_)
B	Maximum fallout, *A*_*tot*,*reg*_, that unmitigated will give rise to at most 20 mSv per year for any age cohort in any year after fallout		
	Newborn male at t = 0	*CED*(70 y) and *CUMLAR*_*WB*_(70 y)	*t*_*b*_ = 0 to *t*_*acc*_ = 70 y
	Newborn female at t = 0		
	30 y old male at t = 0		
	30 y old female at t = 0		
	Fetus	*D*_*Uteru*s_(age(>20 y))	*t*_*b*_ = 0 to *t* = 0.75 y
C	Maximum fallout, *A*_*tot*,*reg*_, that unmitigated will give rise to at most 1 mSv per year for any age cohort in any year after fallout		
	Newborn male at t = 0	*CED*(70 y) and *CUMLAR*_*WB*_(70 y)	*t*_*b*_ = 0 to *t*_*acc*_ = 70 y
	Newborn female at *t* = 0		
	30 y old male at *t* = 0		
	30 y old female at t = 0		
	Fetus	*D*_*Uteru*s_(age(>20 y))	*t*_*b*_ = 0 to t = 0.75 y

*CED(70 y) refers to the 70-y time-integrated cumulative effective dose to a reference person of 70 kg.

Regarding the external exposure component, no snow cover was assumed (*F*_*snow*_ = 1), and this work used the same shielding factor (*f*_*shield*_ = 0.40) and outdoor occupancy factor (*f*_*out*_ = 0.20) as those used in the model calculations by [13]. Furthermore, no remediation was modelled in terms of decontamination to avert external doses; that is, *S*_*decon*t_ = 1. If such measures were to be taken, previous experience shows that a 50% reduction in the external dose can be attained [39], thus giving a value of *S*_*decont*_ around 0.5. Updated methods may, however, result in lower values (to be evaluated in future studies).

For the internal exposure, values of the regional transfer factor of ^137^Cs to humans were selected to mimic the transfer to the general Swedish population after the Chernobyl NPP accident [34]. Therefore, we assumed that initial countermeasures, such as stabling of grazing dairy cattle and distribution of stable iodine to people living in the contaminated areas, were implemented. If these actions are carried out, then even for an internal dose to the thyroid, which is essentially associated with high iodine uptake, it has been shown that the main long-term dose contribution will stem from internal ^134^Cs and ^137^Cs contamination [18]. Thus, the internal exposure from inhalation and intake of radioiodine has been neglected. In addition to these actions, however, no long-term countermeasures were assumed for alimentary transfer of radionuclides to the fictive population; that is, *S*_*aliment*_ is set to 1 in Eqs (1) and (2). It is noteworthy that in Sweden after 1986, the radioecological transfer of ^137^Cs to dairy milk, which is one of the main long-term pathways from ground deposition to urban citizens [36], resulted in maximum regional average values that reached 30 Bq kg^-1^ in areas with an *A*_*tot*,*reg*_ = 45 kBq m^-2^ [40]. Scaling this to the *A*_*tot*,*reg*_ of Scenario A still gives a maximum dairy milk concentration of ^137^Cs, which is beneath the maximum permissible level stated by Euratom [41] of 1000 Bq kg^-1^ in foodstuffs. For the case of more extensive food regulations, such as those in Japan after the Fukushima Dai-ichi NPP accident in 2011, an *S*_*aliment*_ factor closer to 0 than unity would be applied.

It was also assumed that the fictive individuals reside in an area where the local ground deposition of ^137^Cs, *A*_*tot*,*loc*_, is equal to the regional average, *A*_*tot*,*reg*_. If the population in the region is evenly distributed, the average *CED*(70 y) and *CUMLAR*_*WB*_(70 y) values will agree with the individual values for the whole region. However, from Swedish observations, the ratio of the local to regional averaged ground depositions of ^137^Cs can vary over a substantial range, up to a factor of ten within a region [42]. The ratio of local- to regional- (areas in the range of >10,000 km^2^) averaged Chernobyl depositions of ^137^Cs in Sweden ranged from 0.15 to 3.75 ([13] and [43]). The potential effect of this variability on the estimated cumulative effective dose, *CED*(70 y), was studied by [13]. In this study, to account for how the variability can affect the average *CUMLAR*_*WB*_(70 y) values, a simulation similar to Eq (2) was performed to obtain confidence interval estimates of *CUMLAR*_*WB*_(70 y).

## Results and discussion

### Scenario modelling: Detriment and fallout levels at intervention levels

In Scenario A, described in Table 5, the models in Eqs (1) and (2) predict that, with an initial ground deposition of 1 MBq m^-2^ of ^137^Cs, a reference person of weight 70 kg with a sex averaged radioecological transfer of 5.3 (Bq kg^-1^)/(kBq m^-2^) will reach a cumulative effective dose of 123 mSv (Table 6). Furthermore, it was found that an unmitigated fallout will give rise to an initial annual effective dose (including both internal and external contributions) of about 35 mSv y^-1^. When expressing the detriment in terms of time-integrated lifetime attributable risk, *CUMLAR*_*WB*_(70 y), it was found that newborn females at the time of fallout will have a lifetime attributable risk for radiation-induced cancer of 5.4% in this scenario, compared with 3.2% for male newborns. A less pronounced sex difference in *CUMLAR*_*WB*_(70 y) is found for 30 y olds with *CUMLAR* values of 1.2% and 1.0% for females and males, respectively. Furthermore, the *CUMLAR*_*WB*_ values differ greatly between newborns and 30 y olds, and the model predicts a time-integrated lifetime attributable risk that is more than 4.5 times higher for newborn girls than for 30 y old females, for those residing in the affected area more than 70 y.

**Table 6:** Average individual detriment accumulated over 70 y for a resident living in an area affected by a Chernobyl-like NPP remote fallout for newborns and 30 y olds. Detriments are given in terms of cumulative effective dose, *CED*(70 y), attributed detriment using the ICRP (2007) risk coefficients for members of the public (0.05 Sv^-1^), and cumulative lifetime attributable risk of cancer (excluding non-fatal skin cancers) incidence, *CUMLAR*_*WB*_(70 y), for three scenarios described in Table 5.

Scenario: *A*_*tot*,*loc*_ *= A*_*tot*,*reg*_		*CED*(70y) (mSv)	Detriment based on *CED*(70 y)*	*CUMLAR*_*WB*_(70 y)			
	(MBq m^-2^)	70 kg reference person		Newborn		30 y	
				M	F	M	F
*A*	1	123	0.0061	0.032	0.054	0.0095	0.0117
*B*	0.563	69.2	0.0035	0.015	0.026	0.0046	0.0057
*C*	0.028	3.45	1.73·10^−3^	0.77·10^−3^	1.3·10^−3^	0.23·10^−3^	0.28·10^−3^

*Calculated as *CED*(70 y)(mSv)·0.05(Sv^-1^)/1000

When translating the estimated *CED*(70 y) values for Scenario A into the probability of attaining a radiation-induced cancer due to combined external and internal exposures from NPP fallout, a reference person is estimated to attain a value 0.61% per MBq m^-2^ deposition of ^137^Cs. However, when comparing with the corresponding values based on *CUMLAR*_*WB*_(70 y), newborn girls have about 8 times higher estimated probability of cancer incidence per MBq m^-2 137^Cs. This again illustrates, as also pointed out by [12], the importance of considering infants and the youth population in radiological assessments of an NPP fallout. It should be noted, that *CUMLAR*(70 y) for 30 y old, averaged over men and women, are much closer to the detriment calculated from *CED*(70 y), 0.0106 vs 0.0061, respectively. The main difference between these two values can be attributed to the use of a dose and dose rate reduction effectiveness factor in the risk coefficients for late effects, DDREF, of 1.5 by EPA [8], compared with a DDREF of 2 used by ICRP [6].

For Scenario B, it is found that initial *A*_*tot*,*reg*_ values of about 0.56 MBq m^-2^ and higher will lead to an annual effective dose that may exceed 20 mSv for a reference person. Such a ground deposition level will then result in a *CED*(70y) of 69 mSv. The corresponding value for Scenario C, a situation in which the annual effective dose does not exceed 1 mSv is *A*_,*reg*_ = 0.028 MBq m^-2^. The attributed *CUMLAR*_*WB*_(70 y) values are proportionally lower than for Scenario A with respect to initial ^137^Cs ground deposition, *A*_*tot*,*reg*_.

**Fig 3:**
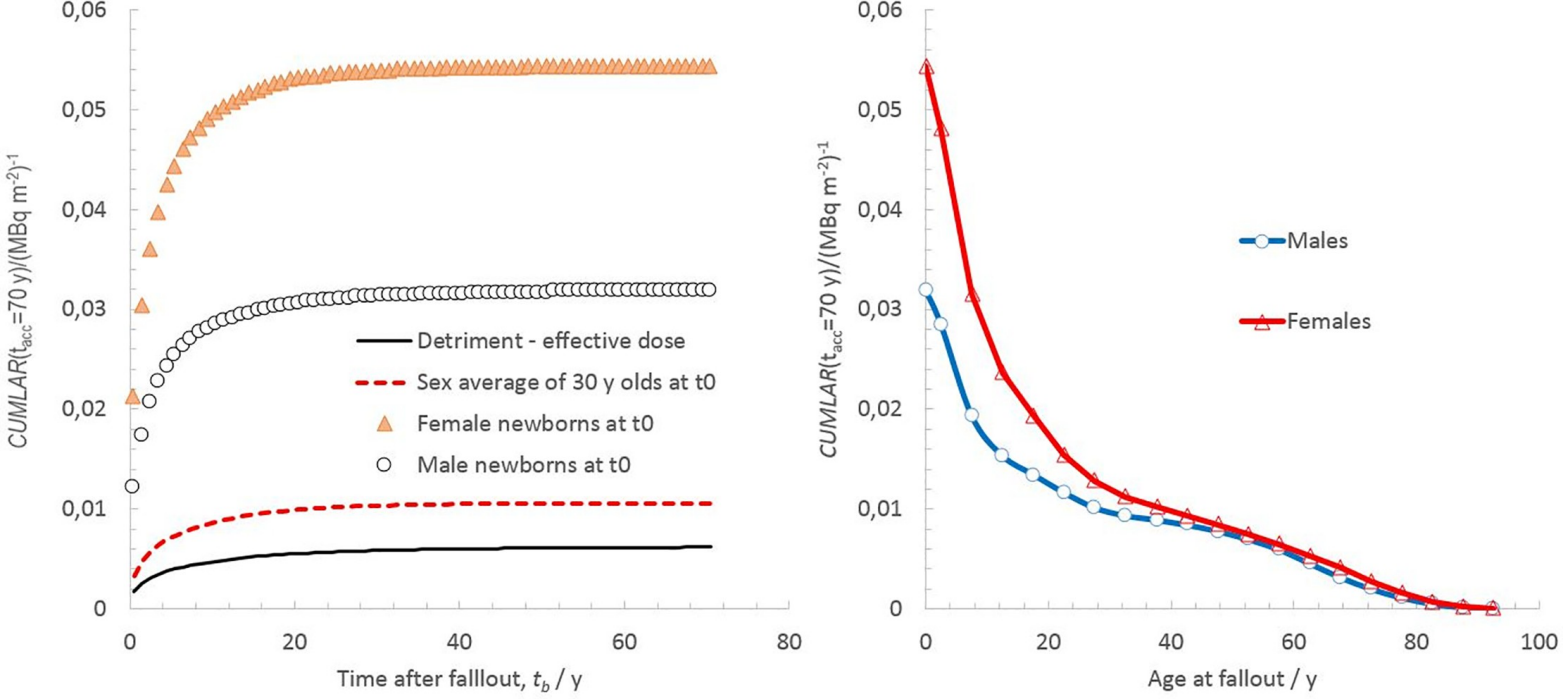
Cumulated lifetime attributable risk of total cancer (excluding non-fatal skin cancers) for *A*_*tot*,*reg*_ = 1 MBq m^-2^. Left: *CUMLAR*_*WB*_(70 y) for newborn females and males at the onset of fallout and the sex-average *CUMLAR*_*WB*_(70 y) for 30 y olds at *t*_*0*_, and the corresponding detriment calculated from effective dose. Right: *CUMLAR*_*WB*_(70 y) as a function of age at the time of fallout for the scenario of *A*_*tot*,*reg*_ = 1 MBq m^-2^ given in Table 5.

Fig 3 (left) plots the *CUMLAR*_*WB*_(70 y) for newborns at the onset of fallout (Scenario A) as a function of time residing in the affected area. The main part of the cumulative detriment up to 70 y is attained faster for newborn females than for the other categories plotted. This is explained by the higher radiation risk coefficients associated with female organs at a young age and is also illustrated by the rapid decline in *CUMLAR*_*WB*,*Females*_(70 y) as a function of age (up to 30 y) at the time of fallout, shown in Fig 3 (right). Furthermore, *LAR*_*WB*_(70 y) appears to plateau over the age span 30–50 y, which combines the effect of exponential decay in internal and external exposures from NPP fallout, as given by Eq (2), and the attributable risk over the long term that is lost due to the limited remaining life expectancy over 70 y for these age cohorts. It is thus again evident that children and young females are of relatively more concern in the event of an NPP fallout compared with other demographic groups.

When comparing the detriment estimates at the age of 30 y in Table 6, it can be seen that the sex-averaged *CUMLAR*_*WB*_(age = 30 y) is still 70% higher than the CED(age = 30y) (see also the plot in Fig 3, left). For cohorts ages 45–50 y (not plotted here), this difference decreased to about 30%. If accounting for ICRP’s use of a dose and dose-rate effectiveness factor (DDREF) of 2 in their age-dependent risk model, instead of a corresponding factor of 1.5 used by EPA [8], the aforementioned difference will even out. The latter age span (45–50 y) is thus the one for which the effective dose detriment and the EPA LAR values will coincide for our three fallout scenarios (A, B, and C).

The estimated *CED*(70 y) per unit ground deposition predicted by the model in Table 6 (~125 mSv/MBq m^-2^ as an average for a reference person of 70 kg for Scenario A is somewhat lower than earlier predictions (e.g., [44]), mostly because our model accounts for the ecological processes that give rise to a relatively short effective ecological half-time of external ^137^Cs exposure (6 y), based on experience from Sweden [20]. If applying a long term half-time in the last term of *r*(*t*) (Table 1), corresponding to a 3% annual decrease in average external dose rate (half-time of 22.8 y) observed in rural Russian settlements [45], our model predicts a *CED*(70 y) of 255 mSv MBq m^-2 137^Cs. Nevertheless, the annual dose rates will be highest the year after the fallout, with a substantial contribution from short-lived radionuclides as described by [21]. The *A*_*tot*,*reg*_(*E*_*max*_ = 20 mSv y^-1^) and *A*_*tot*,*reg*_(*E*_*max*_ = 1 mSv y^-1^) will thus be relatively independent of the long-term ecological half-times of external ^137^Cs exposure assumed in Eq (2).

### Scenario modelling: Impact of different age distributions

Table 7 gives the calculated age-distribution weighted-average individual lifetime attributable risk, *ADWCUMLAR*_*WB*,*sex*_(70 y), in a Scenario A fallout (*A*_*tot*,*reg*_ = 1 MBq m^-2^) for a number of different age distributions. It is seen that the difference in age distributions alone can make population-averaged individual risks vary between 0.010 per MBq m^-2^ for a low-fertility population such as Japan and 0.016 for a high-fertility country such as Egypt. A difference of up to 67% in terms of the population-averaged individual lifetime attributable risk can thus be attributed to varying age distributions.

**Table 7:** Age-distribution-weighted cumulative lifetime attributable risk of cancer incidence (excluding non-fatal skin cancers) over 70 y, *ADWCUMLAR*(*t*_*acc*_ = 70 y), per unit total regional deposition activity of ^137^Cs, *A*_*tot*,*reg*_ (MBq m^-2^), for five types of age distributions taken from the United Nations [38].

Age distribution mean age (y)	*ADWCUMLAR*(*t*_*acc*_ = 70 y) per MBq m^-2^
Japan (46.6)	0.0098
South Korea (41.1)	0.011
USA (39.3)	0.012
India (30.0)	0.015
Egypt (27.7)	0.016

To make the detriment calculations somewhat more realistic, a time-dynamic age distribution could be modelled by also accounting for the birth rate in the affected population. A simple model can be constructed by assuming a constant total fertility rate, *TFR*, and a similar age-dependent mortality rate as in the US population in year 2000 (on which the EPA organ risk coefficients are based on), during time *t*_*ac*c_ = 70 y among the affected population. This approach is analogously to using collective doses, that is, multiplying the estimated individual mean effective dose by the number of individuals in a population over a certain time. This suggested approach, however, means that the total population will change over time depending on changes in *TFR* and age-dependent mortality rate. When comparing the LAR values between different populations having different age distributions, it is more appropriate to sum the LAR values of the sub cohorts (in terms of age and gender), since a population mean value of LAR will be, to a lesser extent, representative of the population dynamics of that particular population. The societal detriment per unit regional ^137^Cs fallout is likely larger in an advanced low-fertility population than for a high fertility developing population, as the loss of even a small fraction of birth cohorts may cause proportionally larger future perturbations of the economic and social sustainability in the low-fertility population.

### Cumulative lifetime attributable risk to offspring of a population living in a contaminated area

According to the model described in Eq (2), assuming the organ dose to the uterus represents the fetal dose, an estimated cumulative absorbed dose averaged over the fetus, 24.5 mGy per MBq m^-2^ deposition of ^137^Cs, is attained if conception and the subsequent gestation occur while residing in that area directly after the fallout. The corresponding fetal doses for Scenarios B and C are 8.1 and 0.41 mGy, respectively. It is beyond the scope of this study to suggest what detriments can be associated with prenatal exposure, but the aforementioned LAR values can still be seen as an upper limit for fetal doses in the described scenarios.

**Fig 4:**
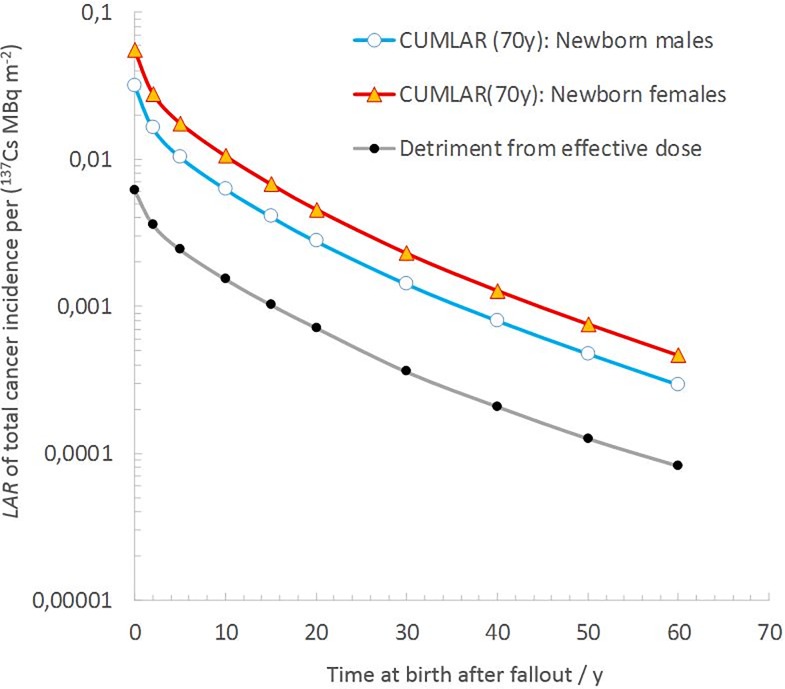
Cumulative lifetime attributable risk of total cancer (excluding non-fatal skin cancers) over 70 y for newborn offspring, born at time *t*_*b*_ after the onset of fallout, for a population living in an area with an initial local- and regional-average ^137^Cs ground deposition, *A*_*tot*,*reg*_, of 1 MBq m^-2^. As a comparison, the detriment from effective dose is also plotted.

If considering the *CUMLAR*_*WB*_ to newborns in the cohort living in the affected areas according to the three scenarios (where there has been no remedial action on external dose and only moderate food restrictions), the maximum *CUMLAR*_*WB*_(*t*_*acc*_ = 70 y) will be the same as for the estimates mentioned in the previous section (Table 6). The *CUMLAR*_*WB*_(*t*_*acc*_ = 70 y) for newborn cohorts as a function of time after fallout is illustrated in Fig 4. Curve fitting of exponential functions to the data using STATISTICA 6.0^TM^ shows that, during the first decade after fallout, the projected detriment for a newborn will decrease with a half-time of 3.0±0.7 y. Ten years after the fallout (*t*_*b*_>10 y), the half-time in estimated *CUMLAR*_*WB*_(70 y) to offspring born in the area is estimated to be 9.0±2.1 y for the following half century. Table 6 and Fig 4 show that, based on the scenarios described, the detriment estimated using the committed effective dose, *CED*(70 y), will consistently underestimate the corresponding detriment for newborns, *CUMLAR*_*WB*_(70 y), based on the BEIR and EPA concept ([7–8]). This underestimation will initially be a factor of 4.5 for newborn boys and gradually decrease to a factor of 3.6 for the newborn cohort offspring 60 y after the fallout. For newborn females, this underestimation will be even larger, with corresponding values ranging from a factor of 8 initially after fallout to 5.8 at 60 y after the fallout.

### Assessment of model uncertainty and comparison with observed data

A preliminary uncertainty assessment of the model was performed in connection with our study. The expression Eq (3) for *CUMLAR*_*WB*_(70 y) for 30 y adult males were coded in calculation software (MATLAB^TM^) and calculated for a total initial regional average ground deposition *A*_*tot*,*reg*_ of 1 MBq m^-2^. The calculations were combined with a simulation engine in which rectangular probability density functions (pdfs) were assigned to the parameters of Eqs (1) and (2). For the parameters *d*_*Cs*_, *Φ*_*K/H*_, *k*_*SEQ*,*K*_(age), *F*_*out*_, *t*_*1*_, *t*_*2*_, *t*_*3*_, *c*_*1*_, *c*_*2*_, *r*_*1*_, *r*_*3*_, *r*_*5*_, and *r*_*7*_, rectangular pdfs that were symmetrically centred around the central values given in Tables 1 and 2 were assigned with a width (central-to-min or max value) ranging from 10%–50% of the central estimate (see Table 8). Wider rectangular pdfs were assigned to *T*_*ag*,*max*_, *FR*, and *F*_*shield*_ (up to almost 100%). The local ground deposition was set equal to *A*_*tot*,*reg*_, but with a log-normal distribution with a geometrical mean of 0.975·*A*_*tot*,*reg*_ and a geometrical standard deviation of 1.252, based on the variance in the local to regional deposition mentioned in [13] and [43]. The body weight was assigned a normal distribution with a 10% relative standard deviation of the mean value for males. The remaining variables in Eqs (1) and (2) were considered fixed in the current simulation. The assigned distributions are largely based on the qualified assumptions presented in [13].

**Table 8:** Parameter values and probability density functions (pdfs) used in the uncertainty assessment. Central estimates of parameters were those used in the standard scenario. The choice of pdfs and assigned distribution parameters are largely based on qualified assumptions (Type B uncertainties), to a large part presented in Isaksson et al. (2019) [13].

Parameter	Value	Unit	Probability distribution
*A*_*tot*,*reg*_	1000	kBq m^-2^	Fixed 1000
*A*_*tot*,*loc*_	1000	kBq m^-2^	Log-normal GM = 0.975, GSD = 1.252
*d*_*Cs*_	0.636	mSv y^-1^/kBq m^-2^	Rectangular (uniform) 0.4452, 0.8268
*Φ*_*K/H*_	0.82	Ratio (dimensionless)	Rectangular (uniform) 0.738, 0.902
*k*_*SEQ*,*K*_ *(age)*	1	Factor	Rectangular (uniform) 0.9, 1.1
*f*_*sex*_	1	Dimensionless	Fixed 1
*F*_*shield*_	0.4	Dimensionless	Rectangular (uniform) 0.1, 0.7
*F*_*out*_	0.2	Dimensionless	Rectangular (uniform) 0.1, 0.3
*w*_*male*_	1	Factor	Normal M = 1 SD = 0.1
*FR*	0.56	Ratio (dimensionless)	Rectangular (uniform) 0.12, 1
*T*_*ag*,*max*_	6.69	Bq kg^-1^/ (kBq m^-2^)	Rectangular (uniform) 0.625, 12.755
*t*_*1*_	1	Y	Rectangular (uniform) 0.5, 1.5
*t*_*2*_	0.75	Y	Rectangular (uniform) 0.5, 1
*t*_*3*_	15	Y	Rectangular (uniform) 10, 20
*c*_*1*_	1	Dimensionless	Rectangular (uniform) 0.8, 1.2
*c*_*2*_	0.1	Dimensionless	Rectangular (uniform) 0.05, 0.15
*S*_*aliment*_	1	Dimensionless	Fixed 1
*S*_*decont*_	1	Dimensionless	Fixed 1
*r*_*0*_	0.96	Dimensionless	Fixed 0.96
*r*_*1*_	36.89025	y^-1^	Rectangular (uniform) 18.45, 55.35
*r*_*2*_	0.1082	Dimensionless	Fixed 0.108
*r*_*3*_	2.447175	y^-1^	Rectangular (uniform) 1.225, 3.675
*r*_*4*_	0.0796	Dimensionless	Fixed 0.0796
*r*_*5*_	0.668408	y^-1^	Rectangular (uniform) 0.334, 1.002
*r*_*6*_	0.0314	Dimensionless	Fixed 0.0314
*r*_*7*_	0.125646	y^-1^	Rectangular (uniform) 0.063, 0.189

The uncertainties in *k*_*SEQ*_ and *k*_*Organ*_ (Table 3), the curve fit constants of *e*_*Cs-134*_(w(age)) and *e*_*Cs-137*_(w(age)), the *LAR* coefficients, and the variation of survival rate (here represented by the overall median life expectancy, *MLE*) were not considered in the uncertainty assessment.

**Fig 5:**
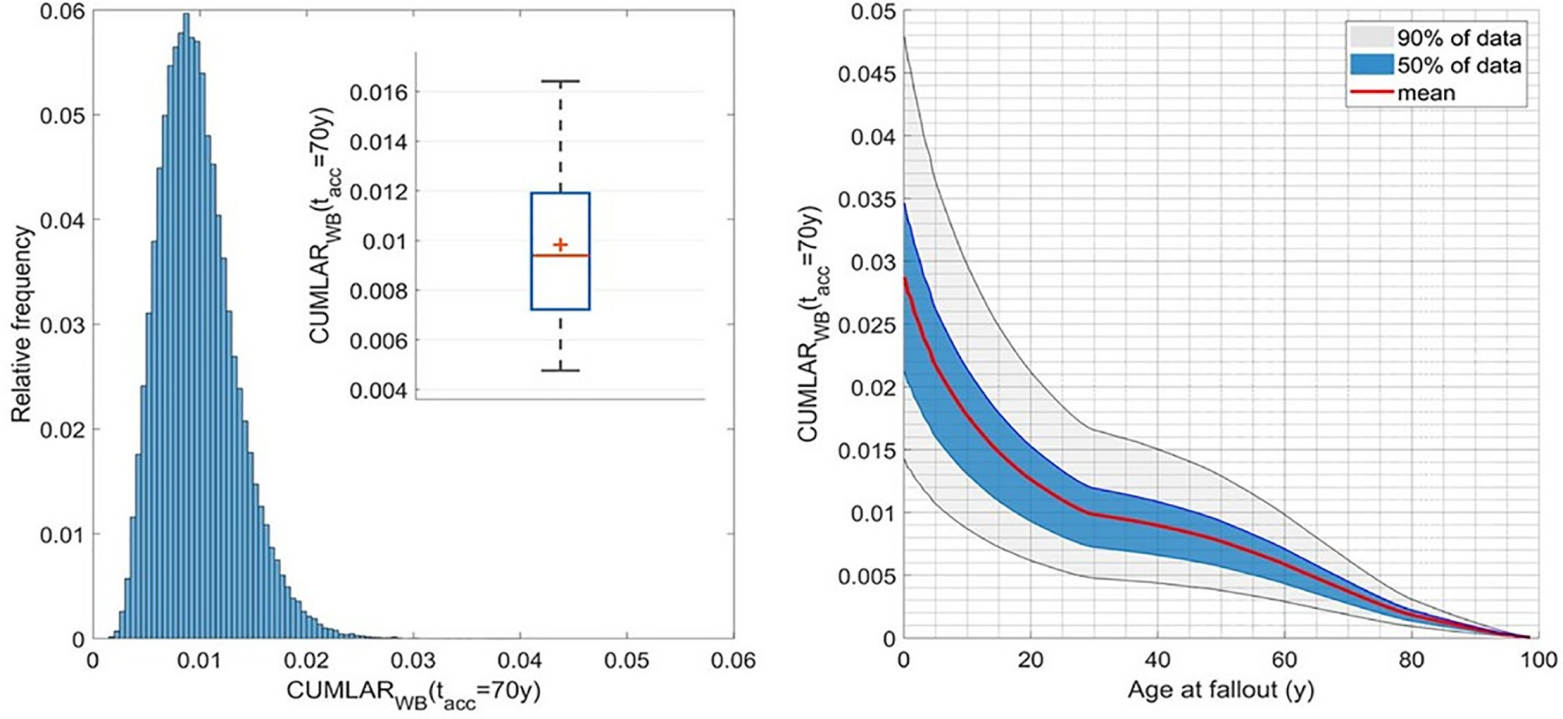
Left: Histogram plot of *CUMLAR*_*WB*_(*t*_*acc*_ = 70 y, *A*_*tot*,*loc*_ *= A*_*tot*,*reg*_ = 1 MBq m^-2^) estimates for adult males (30 y at fallout). Total of 100 kMC runs; results are binned to 5×10^−4^ width bins. In the box plot inset, a red cross represents mean value, the horizontal red line is median value, blue box comprises 50% of data, and whiskers extend from 5^th^ to 95^th^ percentile. Right: Cumulative lifetime probability, *CUMLAR*(*t*_*acc*_ = 70 y), for total cancer in males for an NPP fallout of *A*_*tot*,*reg*_ = 1 MBq m^-2 137^Cs. Grey shaded area comprises 90% of data (from 5^th^ to 95^th^ percentile), blue area comprises 50% of data, and red line represents mean value. 10 000 MC runs for each age point.

The 5^th^ and 95^th^ percentile values in the estimates range from a factor of 0.5 to a factor of 2 of the median value. The histogram plot given in Fig 5 indicates a log-normal-like distribution, but it is in fact a combination of rectangular and log-normally distributed parameters. A continued assessment of the model and its implication for testing different countermeasures, represented by the parameters *S*_*decont*_ and *S*_*aliment*_, needs to be examined in more detail in future studies.

Table 9 provides a comparison of estimates of lifetime attributable risk over the first year (corresponding to *CUMLAR*_*Org*_(age(*t*_*0*_),*t*_*acc*_, *A*_*tot*,*loc*_, *A*_*tot*,*reg*_) in Eq (3)) to residents in some settlements in the Fukushima prefecture, as reported by [9]. The discrepancy between WHO’s and our results are largest for infants (*age*(*t*_*0*_) = 1 y) but, in many cases, are within the aforementioned uncertainty range of the simulated model uncertainty of *CUMLAR*_*WB*_(age(*t*_*0*_),*t*_*acc*_, *A*_*tot*,*loc*_, *A*_*tot*,*reg*_). The discrepancy may partly depend on the *r*(*t*) factor (see Eq (1)) in our model being based on the ^134^Cs/^137^Cs ratio of *FR* = 0.56 in the remote Chernobyl fallout in Sweden [21], compared to *FR* of 1.1 in the Fukushima DNPP release. Further elaboration of our proposed model is therefore necessary in order to extract the extent to which the discrepancies originate from differences in the modelling of particular organ doses, *D*_*org*_ in Eq (2), or from differences in the time pattern of the external dose contribution, *r*(*t*).

**Table 9:** Lifetime attributable risk, *CUMLAR*(*age(t*_*0*_*)*) (LAR*10^−2^), the first year upon fallout in three different Japanese settlements: comparison between WHO estimates [9] and our model estimates. Lifetime attributable risk refers to sum over both solid cancers and leukaemia.

Location	Estimated fallout	Male: *CUMLAR*_*WB*_(age(*t*_*0*_))				Female: *CUMLAR*_*WB*_(age(*t*_*0*_))			
	*A*_*tot*_	1 y old		20 y old		1 y old		20 y old	
	(MBq m^-2^)	WHO	Our model	WHO	Our model	WHO	Our model	WHO	Our model
Naime town	1.5	0.77	1.81	0.409	0.596	1.14	3.16	0.6	0.897
Iitate Village	0.80	0.448	0.97	0.233	0.318	0.663	1.69	0.341	0.478
Katsurao	0.45	0.168	0.54	0.096	0.179	0.249	0.948	0.141	0.269

Generally, cumulative radiation risk estimates and prognoses of associated health consequences over the long term are inherently affected by large uncertainties. The complex features of the LAR concept when accounting for regional specific baseline all-cause mortality rates and its development over time are discussed in more detail in [46]. Ulanowski et al., [46] suggests that long-term radiation-induced detriments can be expressed in terms of incidence of specific diseases, which are considered independent from the effects of radiation on other mortality causes and survival. In doing so, one may also bypass the ambiguity in this work, where the lifetime attributable risk accumulated up to a certain point in time, *t*_*acc*_, during a continuous exposure is conditioned with the probability of not being affected by a radiation-induced cancer up to that given time. If attempting to account for the probability of an individual dying of a cancer induced by the considered radiation exposure, an approximate adjustment to the presented *CUMLAR*_*WB*,*sex*_ values in Table 6 would be the factor (1-*CUMLAR*_*WB*,*sex*_·*p*), where *p* is the gross ratio between lethal and curable cancer cases in that regional cohort. From EPA [8], *p* can be estimated to be 0.40 (males) and 0.41 (females) as a gross estimate for total cancers. For the most sensitive cohort (newborn females) in Scenario A of our model (1 MBq m^-2^ initial fallout of ^137^Cs), this would translate into a downward adjustment of (1–0.054·0.41) = 0.978, and the effect of radiation-induced cancer mortality during the integration time on the accumulated probability of cancer incidence can therefore be considered to be of minor importance.

## Conclusions

Compared to the commonly used risk-assessment model proposed by ICRP using average effective dose, the use of lifetime attributable risk, as elaborated by BEIR [7] and EPA [8], can enable radiological risk estimates from different nuclear power plant release scenarios that are more sensitive to the age and gender of individuals. As was pointed out by [12], there is a large age dependency in terms of lifetime attributable risk, especially for newborn females, with an estimated *LAR*_*WB*_(70 y) per unit ground deposition of ^137^Cs that is more than a factor of 4.5 higher than that for 30 y women. When comparing between the calculated detriment for newborn girls, the LAR-based estimate is almost a factor of 8 higher than the one for the effective dose model (5.4% vs 0.7% (MBq m^-2^)^-1 137^Cs). It should be noted, however, that a part of this difference in estimated detriment is attributed to the use of different DDREF, by ICRP and EPA (2 and 1.5, respectively) in the radiation risk coefficients [6–8].

For a fresh NPP fallout, based on aerial measurements of the ground deposition of ^137^Cs or on corresponding soil sampling surveys, the proposed age- and gender-dependent model can be used to forecast the detriment anticipated if no countermeasures are undertaken. Monte Carlo (MC) simulations indicate an approximate 5% to 95% confidence range of a factor of 2 compared with the central model estimate. If also including the variance in the efficacy of countermeasures, MC simulations of this model can serve as a reference for comparing the averted doses of different countermeasures as a function of age and gender.

We also deduced that the estimated cumulative *LAR*_*WB*_(70 y) for a new cohort will decrease with a half-time of approximately 3 y initially after the fallout, and then of about 10–12 y after 10 y post fallout. This implies that, for the scenarios considered in this study, early countermeasures are more effective in reducing cancer rates attributed to the initial fallout than later mitigation activities. The model can also be used to roughly estimate radiation exposure to the fetus during pregnancy, assuming that the exposure can be represented by the average dose to the uterus for non-pregnant women. It is estimated that at most, about 25 mGy absorbed dose to the fetus is incurred for every 1 MBq m^-2 137^Cs ground deposition (including a standard nuclide vector of associated release products, such as ^134^Cs, ^131^I, ^132^Te, and ^140^Ba), provided that the mother has undergone radioiodine prophylaxis. The LAR concept also enables an estimation of the impact of age distribution in the affected populations. The age-distribution weighted-average cumulative *LAR*_*WB*_, here termed *ADWCUMLAR*, shows that the average individual *CUMLAR*_*WB*_ over 70 y will be about 65% higher in countries with a high proportion of younger inhabitants, like Egypt, compared to, e.g., Japan. The effect of a demographic predominance of young cohorts in the predicted long-term radiological effects after a nuclear fallout may need to be considered in the emergency planning and response activities of countries introducing nuclear power.

Nuclear power countries may have both a highly varying age-specific mortality rate and baseline spectra of various cancer incidences; therefore, the application of gender- and age-dependent radiation risk coefficients from EPA and BEIR may need to be specifically adopted to a given country/region. Any future adjustments in these risk coefficients will also have an impact on the results from our model. Furthermore, the proposed model may be adjusted in terms of radioecological and behavioural modelling to be more accurately applied to areas outside the temperate zones in the Northern Hemisphere. Factors such as outdoor occupancy, the extent to which members of the public will follow food restrictions, and the level to which authorities can actually implement food restrictions and evacuation plans need to be accounted for to obtain more regional-specific values. In addition to these exposure model uncertainties, there are large uncertainties in the dose-risk models in the general populations for various radiation-induced cancers, and its implications have been addressed elsewhere (e.g., [46]). This study, however, serves as an example of the impact that young age has on the radiological effects in connection with a nuclear power plant accident, as well as the contribution from physical models predicting subsequent radiation exposure over the long term, but we believe that a majority of the conclusions found here will still be relevant even when accounting for more regional-specific conditions.

The proposed model is available in the form of a calculation spreadsheet file (LAR_PlosOne_Public_Rev.xlsx) that allows CUMLAR to be computed as a function of age at onset of fallout, gender, and integration time.

## Supporting information

S1 File LAR_PlosOne_Public_Rev.xlsx. A calculation spread sheet for tentative calculations of cumulative effective dose to a reference person, *CED*(t_acc_), cumulative organ absorbed dose, *D*_*org*_(t_acc_) and cumulative life-time attributable risk to the organ, *CUMLAR(*t_acc_), as a function of time considered for exposure to ground deposition of humans of gender (M/F) and age in the affected area.
(XLSX)
